# WHO Global Research Agenda for Hand Hygiene Improvement in Health Care: a Delphi Consensus Study

**DOI:** 10.1017/ice.2025.32

**Published:** 2025-03-20

**Authors:** Benedetta Allegranzi, Ermira Tartari, Claire Kilpatrick, Julie Storr, Nita Bellare, João Bana, Ana Flávia Santos, Sarah Charnaud, Anna Laura Ross, Mitchell J. Schwaber, Didier Pittet, on behalf of the WHO Technical Advisory Group on Hand Hygiene Research, Yewande Alimi, Jameela AlSalman, Abdallah Al Qayoodi, Fernando Bellissimo-Rodrigues, John Conly, Prakash Ghimire, M. Lindsay Grayson, Mushtuq Mohammad Husain, Paul Hunter, Kushlani Jayatilleke, Samuel Kaba, Pamela Yew Fong Lee, Margaret Leong, Fernanda C. Lessa, Maryanne McGuckin, Geeta Mehta, Inmaculada Fernandez Moreno, Peter Nthumba, Folasade Ogunsola, Molly Patrick, Diamantis Plachouras, Jacqui Reilly, Kyla Smith, Miranda Suchomel, Seto Wing Hong

**Affiliations:** Africa Centres for Disease Control and Prevention, Ethiopia; Arabian Gulf University, Bahrain; Ministry of Health, Oman; University of São Paulo, Brazil; University of Calgary and Alberta Health Services, Canada; Tribhuvan University, Nepal; University of Melbourne, Australia; Institute of Epidemiology, Disease Control and Research, Bangladesh; University of East Anglia United Kingdom of Great Britain and Northern Ireland; Sri Jayewardenepura General Hospital, Sri Lanka; Institutional Care Division, Ghana Health Service, Ghana; Sarawak General Hospital, Ministry of Health, Sunway University, Malaysia; Pacific Community, Fiji; International Infection Control Branch, Centers for Disease Control and Prevention, Atlanta, USA; McGuckin Methods International, USA; IPC independent consultant, India; Hospital Parc Taulí Sabadell, Spain; AIC Kijabe Hospital, Kenya; Infection Control Africa Network, Nigeria; Centers for Disease Control and Prevention, USA; European Centre for Disease Prevention and Control, Sweden; Glasgow Caledonian University, United Kingdom of Great Britain and Northern Ireland; WaterAid, United Kingdom of Great Britain and Northern Ireland; Medical University of Vienna, Austria; School of Public Health, Hong Kong Special Administrative Region, China; 1 Infection Prevention and Control Unit and Hub, Department of Integrated Health Services, World Health Organization, Geneva, Switzerland; 2 Faculty of Health Sciences, University of Malta, Msida, Malta; 3 Decision Eyes, Lisbon, Portugal; 4 CEGIST, Instituto Superior Técnico, Universidade de Lisboa, Lisbon, Portugal; 5 Research for Health department, World Health Organization, Geneva, Switzerland; 6 National Center for Infection Control, Israel Ministry of Health, Jerusalem, Israel; 7 Faculty of Medicine, University of Geneva, Geneva, Switzerland

**Keywords:** infection prevention and control, healthcare-associated infections, antimicrobial resistance, hand hygiene, research prioritization, Delphi process

## Abstract

**Objective:**

To identify global research priorities for improving hand hygiene in healthcare settings and develop a 2023-2030 research agenda to guide funding, coordinate research, promote investment, and inform policy for enhanced healthcare quality and safety.

**Design:**

Expert consensus study using a modified Delphi process.

**Participants:**

A 105-member panel of international hand hygiene experts including the World Health Organization (WHO) Technical Advisory Group of Experts on Hand Hygiene in Healthcare representing all WHO regions and World Bank income levels.

**Methods:**

The research priorities were identified through a multiphase approach including a meta-review to establish knowledge gaps and inform initial priorities, followed by expert consultations using a modified Delphi process. 192 preliminary priorities were included in a two-round Delphi survey. Experts rated each priority in the first round, and then reviewed and adjusted responses based on the panel’s aggregated, anonymous responses in the second round. Ratings were collected on a five-point Likert scale. Consensus was defined as a combined ‘strongly agree’ and ‘agree’ frequency of at least 70%.

**Results:**

Consensus was achieved for 178 of 192 priorities (92.7%), categorized into six domains: system change; training and education; evaluation and feedback; reminders and communications; institutional safety climate; and hand hygiene improvement impact on healthcare-associated infections and antimicrobial resistance. Of these, 121 priorities reached >80 % consensus. The Delphi process, maintained a 92% response rate over two rounds.

**Conclusions:**

A structured consensus process yielded a research agenda to address gaps in hand hygiene improvement, supporting enhanced healthcare quality and safety globally.

## Introduction

Hand hygiene is one of the most effective measures to prevent microbial transmission and infection in healthcare.^1,2^ Over three decades, key milestones have included replacing traditional handwashing with soap and water with alcohol-based handrub (‘system change’) and implementing a multimodal strategy to influence healthcare workers’ behavior. This innovation, pioneered at the Geneva University Hospitals^3^ spurred international research^4^ and led the World Health Organization (WHO) to develop and test the multimodal hand hygiene improvement strategy (MMIS).^5–7^

Building on scientific evidence, WHO, the United States Centers for Disease Control and Prevention (CDC) and the Society for Healthcare Epidemiology of America (SHEA) issued comprehensive hand hygiene recommendations.^1,2,8,9^ Despite being a simple measure, the 2009 WHO guidelines^1^ highlighted research gaps, marking the beginning of an era of intense investigation into infection prevention and control (IPC). In 2017, international experts revisited and updated the 2009 research agenda.^4^

One major advancement was the WHO MMIS, recognized as the gold standard for promoting behavioral change in hand hygiene at the point of care.^5,6^ The MMIS has since been adapted for broader IPC use and incorporated as a core component of WHO’s recommendation for effective IPC programs.^5,10^ WHO global surveys (2011, 2015, 2019) investigating the status of hand hygiene programs according to a framework based on the MMIS ^11,12^ reported an intermediate level of hand hygiene implementation.^12,13^

This study aims to establish an international consensus on critical research priorities for advancing hand hygiene in healthcare (2023-2030). The consensus seeks to coordinate efforts among stakeholders, funders and researchers to address urgent evidence gaps.

## Methods

**Figure 1:**
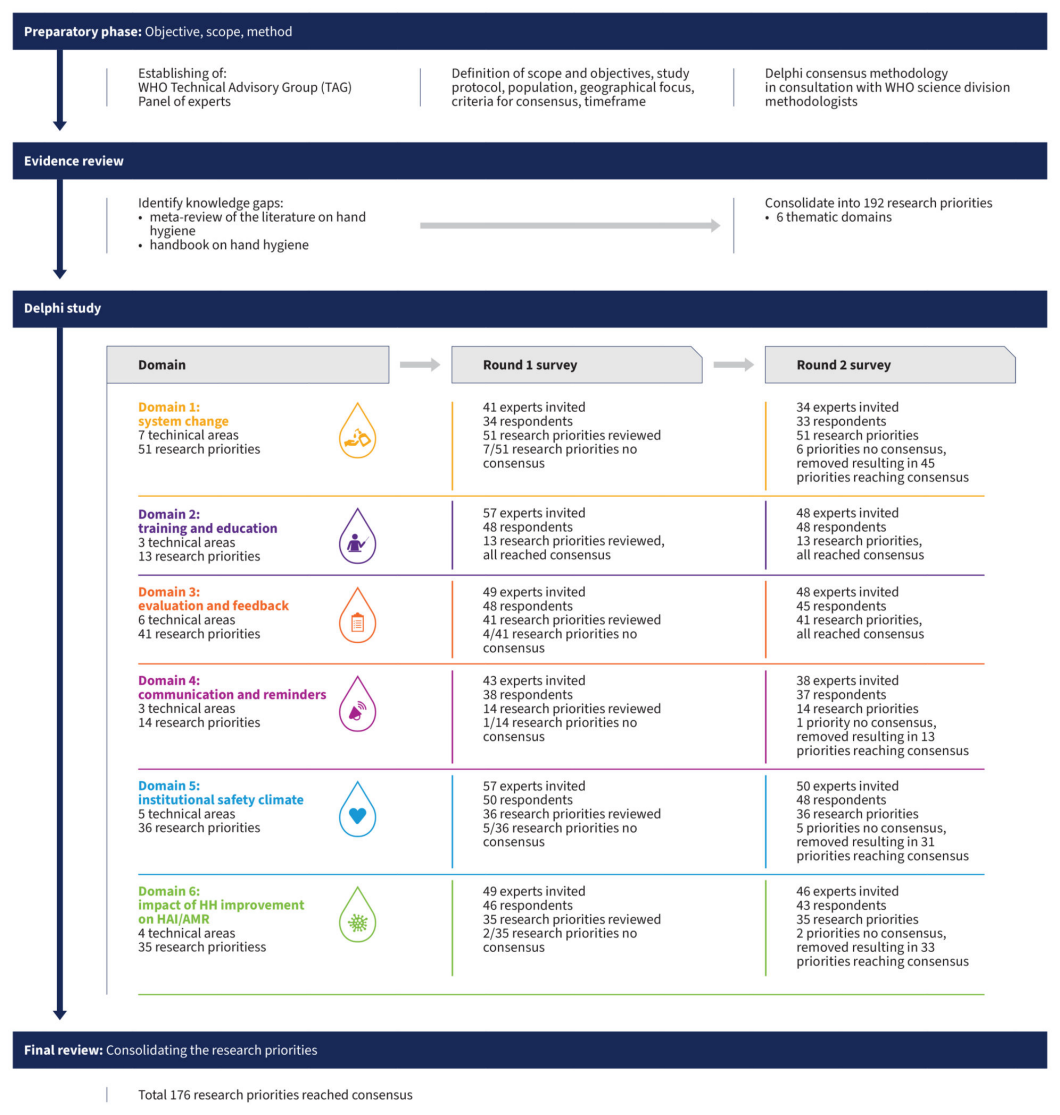
Flowchart of the study process for identifying the 2023-2030 research agenda for hand hygiene in healthcare. Footnote: HH, hand hygiene; HAI, healthcare-associated infection; AMR, antimicrobial resistance This process was carried out between March 2022 and February 2023, using the Delphi consensus methodology to identify research priorities for hand hygiene in healthcare settings. The research agenda for 2023-2030 was developed based on input from an international panel of experts, in consultation with WHO methodologists.

A multiphase approach (Fig. 1), including a Delphi consensus process^14–16^ was used to determine research priorities on hand hygiene in healthcare. The study protocol adhered to recommendations from the Conducting and REporting of DElphi Studies (CREDES).^17^ Ethical approval was obtained from WHO’s Ethics Review Committee (ERC #0170).

A meta-review and an analysis of the research gaps identified in ‘Hand hygiene: a handbook for medical professionals’^4^ building on the WHO hand hygiene guidelines^1^ provided the basis for preliminary research priorities categorized into six domains: (1) system change; (2) training and education; (3) evaluation and feedback; (4) reminders and communications; (5) institutional safety climate; and (6) the impact of hand hygiene improvement on antimicrobial resistance and healthcare-associated infections (HAIs.)

### Participants

A Technical Advisory Group (TAG) on Hand Hygiene in Healthcare Research was established,^18^ comprising 27 experts across six WHO regions and World Bank economic income levels^19^; gender balance was also ensured. Additional experts were recruited through literature searches, WHO networks and regional offices. Experts were assigned to domain-specific working groups to refine research priorities.

### Delphi Procedure

A modified Delphi process comprising a predetermined number of survey rounds, was used to establish the final research priorities for hand hygiene. Delphi studies were conducted for each of the six domains (Fig. 1), with two rounds of online surveys administered between March 2022 and February 2023. Surveys remained open for 10 days, with two reminders sent for completion. Results were analyzed to produce a final list of research priorities categorized into six thematic domains, with priorities ranging from 14 and 52 per domain.

Participants attended an online meeting before each Delphi survey to receive instructions and review preliminary research priorities. The working group chair and facilitator led the discussions to refine priorities by rephrasing, removing duplicates, and merging similar topics. Feedback informed the final list of research priorities for inclusion in round 1. Surveys were administered via the piloted Welphi platform, ^20^ ensuring clarity and ease of use.

#### Round 1

Participants were provided access to a web-based questionnaire containing the identified research priorities, and they rated each one according to the research priority setting dimensions (Table 1) and their assessment of the need for further evidence. A 5-point Likert scale was used, ranging from 1 (‘totally disagree’) to 5 (‘totally agree’), with an additional option of ‘unable to rate’. The primary objective was to achieve consensus on the research priorities to be included in the final hand hygiene research agenda for each of the six domains. Survey respondents were also invited to provide comments, including suggestions for revising the proposed priorities and proposing additional items for consideration (Supplementary Material, Appendix A). Consensus was defined as ≥70% of participants agreeing or strongly agreeing with a research priority statement, a threshold considered appropriate in previous Delphi studies.^21–23^ ‘Disagreement’ was defined as occurring when 35% or more of the responses fell within the extreme ranges of the Likert scale. For each research priority statement, consensus proportions were calculated based on the number or percentage of respondents, excluding the ‘unable to rate’ responses.

#### Round 2

Participants who completed round 1 were invited to participate in round 2, which focused on re-evaluating the research priorities that had not reached a consensus in the first round. Round 1 results were shared with participants, including a visual representation of the distribution of scores from all respondents, alongside their individual score, using the same rating scale. Participants were asked to consider the responses of others and review their ratings, with the option to either confirm o them.

### Statistical Analysis

Survey data were analyzed using SPSS version 25 (IBM, Armonk, NY, USA). Descriptive statistics summarized participants’ responses, with consensus calculated for each priority. Ranking was based on mean scores and standard deviations calculated for each item.

## Results

Following a meta-review of the literature and expert consultations, a preliminary list of 192 research priorities was developed. Through the six Delphi studies, 14 statements did not reach the predefined consensus level and were eliminated, resulting in a final list of 178 priorities (Table 2). A panel of 105 international hand hygiene experts, including the WHO Technical Advisory Group of Experts on Hand Hygiene in Healthcare representing all WHO regions and World Bank income levels participated. Overall, there was a high level of agreement among respondents, with 121 research priorities reaching >80% consensus.

Participants (n=105) came from multiple disciplines, including IPC (72%), infectious diseases (45.6%), microbiology (28%), patient safety and quality (49%), and research and academia (45.6%); 40% had over 15 years of experience in their respective fields. A high response rate (92%) was achieved on average for the Delphi surveys. There was a higher representation of participants from high-income countries 57/105 (54%) than low-income countries 27/105 (26%) (Table 3). The rate of “unable to rate” responses across all rounds and domains was 1.85%, calculated as the proportion of “unable to rate” answers (3.42) relative to the total number of responses (185).

### System Change

Of the 41 experts invited to participate in round 1 of this domain, 34 (83%) responded and were invited to round 2. Of these, 33 (98%) responded. Fifty-one research priority statements were initially included and classified in the following technical areas within this domain: (1) general system change issues/enabling environment; (2) surgical hand preparation; (3) hand hygiene agents; (4) reactions to hand hygiene agents; (5) barriers/enablers to hand hygiene compliance; (6) the skin microbiome; and (7) dynamics of hand transmission. Finally, 45 statements reached consensus among respondents, with 24 (53.3%) achieving a consensus level of >80% (Supplementary Material, Appendix B) and six were excluded (Supplementary Material, Appendix C). The top three research priorities with the highest consensus are presented in Table 4.

### Training and Education

Of the 57 experts invited to participate in round 1 of this survey item, 48 (87%) responded. Fifteen research priority statements were initially included and all achieved consensus in round 1, including 13 (71%) with a level of consensus >80% (Supplementary Material, Appendix B). Research priority statements were classified in the following technical areas within this domain: (1) training and education strategies; (2) training and education of IPC specialists, healthcare workers and other personnel; and (3) hand hygiene in specific patient populations and situations. The top three research priorities with the highest consensus are presented in Table 4.

### Evaluation and Feedback

Of the 49 experts invited to participate in round 1 of this survey item, 48 (98%) completed the survey and were invited to round 2. Of these, 45 (94%) responded to the questionnaire. Forty-one research priority statements were included and classified into the following technical areas within this domain: (1) compliance with hand hygiene best practices; (2) physicians and hand hygiene; (3) monitoring hand hygiene performance; (4) performance feedback; (5) monitoring within your institution; and (6) external regulation and evaluation. All proposed statements reached consensus through the two survey rounds; 25 statements (61%) reached a consensus level of >80% (Supplementary Material, Appendix B). The top three research priorities with the highest consensus are presented in Table 4.

### Reminders and Communications

Of the 43 experts invited to participate in round 1 of this survey item, 38 (88%) responded and were invited to round 2. Of these, 37 (97%) responded to the questionnaire. Fourteen research priority statements were initially included in the survey and classified in the following technical areas within this domain: (1) hand hygiene promotion; (2) hand hygiene marketing approaches; and (3) hand hygiene campaigns and communication. Finally, 13 statements reached consensus among respondents, with 9 (69.2%) achieving a consensus level of >80% (Supplementary Material, Appendix B), and one was excluded (Supplementary Material, Appendix C). The top three research priorities with the highest consensus are presented in Table 4.

### Safety Climate/Culture Change

Of 57 experts invited to participate in round 1 of this survey item, 50 (88%) responded and were invited to round 2. Of these, 48 (96%) responded. Thirty-six research priority statements were initially included in the survey and classified in the following technical areas within this domain: (1) general aspects of safety climate/culture change and hand hygiene; (2) personal accountability for hand hygiene; (3) leadership; (4) patient participation and empowerment; and (5) religion and tradition. Finally, 31 statements reached consensus among respondents, with 22 (71%) achieving a consensus level of >80%, and five were excluded (Supplementary Material, Appendix C). The top three research priorities with the highest consensus are presented in Table 4.

### Impact of Hand Hygiene on HAIs and the Transmission of Antimicrobial Resistance

Of 49 experts invited to participate in round 1 of this survey item, 46 (94%) responded and were invited to round 2. Of these, 43 (93%) responded to the questionnaire. Thirty-five research priority statements were initially included in the survey and classified in the following technical areas within this domain: (1) effect of hand hygiene on transmission, colonization and/or infection; (2) hand hygiene impact in specific settings; (3) hand hygiene impact in settings with limited resources; (4) the economic impact of improved hand hygiene. Finally, 33 statements reached consensus among respondents, with 30 (85.7%) achieving a consensus level of >80%, and two were excluded (Supplementary Material, Appendix C). The top three research priorities with the highest consensus are presented in Table 4.

## Discussion

The establishment of a prioritized 2023-2030 research agenda for hand hygiene in healthcare settings is a crucial step toward addressing the persistent gaps in IPC evidence. To the best of our knowledge, this is the only existing comprehensive research agenda that has identified 178 research priorities for hand hygiene in healthcare through a multiphase, structured consensus approach using a Delphi process and achieving consensus among 105 international experts from multiple disciplines. A high level of consensus (>80%) was achieved on 121 research priorities, underscoring critical areas needing attention and providing a roadmap for future research and practice improvement. Recently, other research agendas have been published, including those focused on multidrug-resistant organisms transmission prevention, antimicrobial resistance, antibiotic stewardship and cleaning, and environmental hygiene, thus highlighting the growing emphasis on systematic approaches to address critical knowledge gaps and healthcare challenges.^24–27^

Among the highly prioritized research areas in the domain ‘evaluation and feedback’, recommendations with over 85% consensus highlighted the role of unobtrusive monitoring methods such as electronic monitoring compliance systems (87%). This suggests a strong need to integrate advanced monitoring technologies to provide real-time feedback and improve hand hygiene practices. Little is known about the long-term advantages and unintended consequences of electronic monitoring systems and further research is recommended.^2,28^

In the ‘system change’ domain, there was a very high consensus (91%) on the importance of legislative and policy mechanisms for sustaining system change, highlighting the necessity for robust regulatory frameworks to support hand hygiene initiatives. Consensus was also reached on the critical need to develop new international standards for the efficacy of hand hygiene products and to explore the economic impact of local versus imported hand hygiene formulations. In addition, evaluating the cost-effectiveness of the WHO MMIS achieved a high consensus (91%), highlighting the need for studies on cost-effectiveness and economic analyses to evaluate the financial implications of hand hygiene interventions that can further support resource allocation and justify investment in hand hygiene improvements across all resource settings.^29–31^

Important gaps identified the need to understand the role of the skin microbiome and hand transmission dynamics and how these biological factors influence overall hand hygiene effectiveness. The high level of agreement on the influence of human factors, such as the placement of sinks and the role of glove use, indicates a growing recognition of the need to design hand hygiene systems that are both user-friendly and effective in preventing contamination.^32^

In the ‘training and education’ domain, there was strong agreement (95% consensus) that more research is needed to evaluate the effectiveness of train-the-trainer approaches across various settings, reflecting their potential to standardize hand hygiene training and to ensure continuous capacity-building and improvement.^33,34^ These recommendations will help shape future research by focusing on developing scalable and sustainable hand hygiene interventions, emphasizing the role of technology and structured training programs.^35^

Several research priorities that achieved a high consensus (>90%) highlighted critical knowledge gaps. For example, the high level of agreement from the expert group in investigating the relationship between hand hygiene compliance and the reduction in HAI incidence underlines the gap in this area across countries, which demands robust studies to establish causal inferences and quantify the impact of hand hygiene on HAI incidence, thereby providing empirical evidence to inform policy and practice.

The final research agenda emerged with two important cross-cutting themes. First, it stressed the potential role of technology, from electronic monitoring to innovative tools for training and education.^35^ Artificial intelligence is poised to play a transformative role in training, offering personalized learning experiences, adaptive feedback, and real-time performance assessment, which could enhance hand hygiene education and compliance monitoring.^36^ This finding reflects an opportunity to leverage technological advancements to enhance IPC measures. The second theme was the relationship between a positive institutional safety climate and improved hand hygiene practices. Research in this area, especially the influence of leadership and organizational culture, will be crucial in fostering healthcare organization environments where hand hygiene practices are consistently prioritized.^4,37^

The research agenda was designed to be global in scope, addressing shared challenges across income settings while placing particular emphasis on bridging knowledge gaps in low-income settings. Experts were asked to rate priorities based on standardized dimensions—impact/significance, cost-effectiveness, and feasibility, ensuring consistency across evaluations while allowing for the incorporation of contextual realities. Although the agenda is global, it is not prescriptive but adaptive, encouraging local adaptations to align with specific country contexts and needs. For instance, while advanced technological interventions are highlighted, their implementation in low-income countries could focus on low-cost, scalable adaptations. Similarly, policy-related priorities may be tailored to reflect variations in healthcare infrastructure and governance systems. Adaptation ensures that the agenda remains relevant and actionable across diverse settings.

### Strengths and Limitations

The Delphi process is a strength of our study as it involved international experts from diverse backgrounds and locations worldwide. It also preserved anonymity between the participants and their feedback during the study phases and rounds, thus enhancing the credibility of all responses. The Delphi study was informed by a meta-review, and a high response rate was achieved in the surveys, ensuring that the final consensus on the future research agenda for hand hygiene was widely accepted. The literature provides varying thresholds for acceptable agreement among participants in Delphi studies. We preselected a threshold of 70% in each round, which was within the range of agreement rate values suggested in the literature (51%-100%).^23,38–41^

Our study has some limitations. Despite efforts to invite internationally-renowned experts with extensive experience from diverse geographic regions and income settings, some regions remained underrepresented, and most respondents were from high-income countries. This composition may have introduced bias, as priorities identified by participants from high-income settings may not fully align with the needs or applicability to low-income settings.

While we achieved a reasonable spread of countries, further diversifying the expert panel could have mitigated this limitation. Nonetheless, the Delphi method carries an inherent risk of overlooking certain areas due to the subjectivity of expert opinions and variations in thresholds for acceptable agreement in Delphi studies. These factors may have resulted in some important areas being missed or underemphasized.

A potential limitation is the high level of agreement among the expert panel, which may have made it challenging to rank the research priorities effectively. However, this also highlights the broad consensus on key research areas, allowing different research groups to focus on distinct aspects. Finally, internal biases among experts may have influenced the choices and cannot be ruled out.

In conclusion, we believe that this prioritized research agenda of 178 items for hand hygiene in health care offers a comprehensive guide for healthcare organizations, researchers, clinicians, policy-makers and funding bodies across various resource settings. By focusing on areas such as advanced monitoring technologies, legislative support, structured training programmes, and addressing critical knowledge gaps through transdisciplinary research, the agenda aims to globally enhance the quality and safety of healthcare delivery. The WHO IPC global strategy,^42^ action plan and monitoring framework^43^ identify the development of a global research agenda for IPC as a key action to support countries in creating their national research agendas. This Delphi study provides a legitimacy and enhanced value to this work and is intended to help countries to meet the global targets of national research agendas. ^43^

## Supplementary Material

Supplemental File

## Figures and Tables

**Table 1:** Research priority setting dimensions

**Impact/significance**: It is important to conduct this research as more evidence is likely to have a significant impact on strengthening an organization’s safety climate and hand hygiene improvement.
**Cost-effectiveness**: Conducting research on this designated research priority/intervention is cost-effective.
**Feasibility:** Can research bridge the gap? To what extent is it practical and feasible to undertake research on this priority (eg, considering the resources needed, technical challenges to be overcome, and support required)?

**Table 2:** Final 2023-2030 research agenda for hand hygiene in healthcare categorized into six thematic domains following expert consensus

	Research statement
**System Change**	
General system change – enabling environment	The approaches or interventions required to facilitate sustained system change in the context of the multimodal improvement strategy (MMIS). The role of legislation/policy and mechanisms for enforcement on sustained system change and the impact on hand hygiene compliance. The prevailing unintended consequences on the healthcare system, transpiring from sustained system change efforts in the context of the MMIS. The factors in the enabling environment that determine hand hygiene station availability, quantity, distribution and location. The relationship between hygiene service ladder levels as defined by the Joint Monitoring Program (basic, limited and no services) and hand hygiene compliance. The relationship between hygiene service ladder levels as defined by the Joint Monitoring Program (basic, limited, and no services) and healthcare-associated infections (HAIs). Accessibility policies/standards for health workers, patients (including children) and visitors that address equity, diversity and inclusivity, and the relationship with hand hygiene compliance. To assess the role of public health emergencies of national/international concern on availability of hand hygiene infrastructure, materials and equipment and the impact on hand hygiene compliance.
Surgical hand preparation	The optimal hand hygiene technique for surgical hand preparation to ensure improved antimicrobial activity as well as tolerability to agents. The role of powdered gloves and their effect on the quality of surgical hand preparation. The availability of potable/drinking water and its impact on compliance with surgical hand preparation. The role of hand hygiene agents’ tolerability and acceptability on surgeons’ skin and the relationship with adherence to surgical hand preparation. Alcohol-based surgical hand preparation compared to traditional hand scrubbing and the implications for microbial load, water consumption and waste production.
Hand hygiene agents	Economic evaluation of local alcohol-based handrub (ABHR) production versus local market availability. The factors that facilitate availability of locally-produced, efficacious and cost-effective hand hygiene agents. The development of international standards and norms on assessing antimicrobial activity of hand hygiene agents. The development of (new) international standards and norms and their influence on agent availability in low- and middle-income countries. Hand hygiene agents’ efficacy in removing a range of organisms from health worker hands, including *Clostridioides difficile* spores and respiratory viruses, and the impact on transmission and HAIs. The role of emollients and agents in gel or foam form on the efficacy, tolerability and acceptability of ABHR formulations. The predictive model (including concentration and purity) that can be used in the formulation of new effective, acceptable and tolerable hand hygiene agents. The impact of refilling bottles with ABHR on the efficacy of the agent.
Reactions to hand hygiene agents	The impact of dermal harm from hand hygiene agents in a range of settings and skin types (as reported to occupational health) and the relationship with hand hygiene compliance. The impact of dermal harm from hand hygiene agents in a range of settings and skin types (as reported to occupational health) and the relationship with healthcare worker work.
Barriers/enablers to hand hygiene compliance	The use of gloves and the influence on hand hygiene adherence and pathogen transmission. The correlation between workload: staffing ratio, the built environment and hand hygiene compliance including during outbreaks. The factors that influence the selection of ABHR (eg, ambient environment/temperature and humidity on drying time, ease of spread on hands, and stickiness of different handrubs/staff comfort, cost) and the correlation with hand hygiene compliance. The factors that influence the use of ABHR and the relationship with hand hygiene compliance. Hand drying options and the impact on organism removal and hand hygiene compliance. The location of hand hygiene facilities, both sink and ABHR placement (agent: bed ratio) and the relationship with hand hygiene compliance. The role of sink placement and water splashes and the relationship with hand hygiene adherence and HAI in intensive care units, neonatal units. The role of automated dispensers, their placement and their influence on hand hygiene compliance. The role of human factors on hand hygiene compliance.
The skin microbiome	Development of sample collection and testing methods to understand the dynamics of hand microbiota. The relationship between the quantity of gastrointestinal and respiratory viruses present in the environment and the need for hand hygiene action to interrupt environmental contamination and transmission. The role of hand hygiene agents and moisturizers on the skin microbiome/health. The role of hand microbiota and the impact on infection acquisition in a range of commonly encountered interventions, eg, long lines, wounds, etc. The role of commensal hand microbial flora in resisting colonization by commonly known healthcare pathogens. The extent of colonization from healthcare worker hand microbial flora and the correlation with HAI in pre-determined hospitalized patients. The role of the skin microbiome across different age groups and the correlation with microorganism transmission and HAIs. The minimum level of potentially pathogenic organisms that need to be present on hands to cause transmission and HAI in a range of settings. To develop standardized methods for determining hand contamination from a pre-determined list of touch points in clinical settings.
Dynamics of hand transmission	The role of fomite contamination in hand contamination with different microbes, the potential for transmission and the correlation with HAI. The minimum infectious dose of potentially pathogenic organisms on environmental contamination and the impact on the times for hand hygiene. The role of the built environment and ergonomics/human factors in hand recontamination. The impact of glove removal on hand (re)contamination from pathogenic organisms in a range of settings.
**Training and Education**	
Training and education strategies	To identify the optimal educational methods to improve health worker understanding of the dynamics of transmission at the point of (patient) care. To evaluate the impact of different hand hygiene training and educational strategies (face-to-face and virtual, participatory, team and task-based strategies that are participatory and include bedside and simulation) on the knowledge and skills (eg, appropriateness of hand hygiene technique) of health workers across the levels of the health system (primary, secondary, tertiary and long-term care). To determine the effectiveness of the train-the-trainer approach on sustained hand hygiene improvement across settings and populations. To evaluate the impact of hand hygiene training strategies that use apps, technology, gamification, smart phones, simulation, role play, use of audiovisual, practical training on sustained hand hygiene compliance improvement across different settings and countries. To determine the influence of different training strategies on appropriate glove use and the performance of hand hygiene at the right moment by health care workers.
Training and education of IPC specialists, healthcare workers and other personnel	To determine the best approaches for training and educating health workers in specialized settings (included but not limited to intensive care, anesthesiology, surgical departments, dialysis, long term care, ambulatory care, neonatal/pediatric). To evaluate optimal methods to train and educate health workers to become champions and role models for hand hygiene in the context of their area of specialty. To identify the best approaches to educate patients and family members in hand hygiene improvement efforts while taking social and cultural context into account. To evaluate the best approaches for training and education in resource poor settings. To determine the optimal strategy to provide hand hygiene training and education in local language while minimizing translation biases.
Hand hygiene in specific patient populations and situations	To assess the impact of in-service training on hand hygiene on health worker behavior (hand hygiene compliance) in the short- and long-term. To evaluate the optimal training and education approach for health workers undertaking hand hygiene monitoring. To assess the effectiveness of a train-the-trainer session in hand hygiene training and education strategies on the prevention of device-associated infections. To assess the effectiveness of a train-the-trainer session in hand hygiene training and education strategies on the incidence of drug-resistant organisms’ cross-transmission in healthcare facilities. To evaluate the impact of education strategies on hand hygiene knowledge across different audiences (eg, managers versus healthcare workers).
**Evaluation and Feedback**	
Compliance with hand hygiene best practices	Influence of specific cultures and social and religious environments on hand hygiene compliance by different healthcare workers. Use of data on barriers and predictors of hand hygiene compliance during feedback to improve hand hygiene action. Caregivers’ hand hygiene compliance in during care provided in the community (including home care). Caregivers’ hand hygiene compliance in during traditional medicine interventions. Caregivers’ hand hygiene compliance during care in acute healthcare facilities. The role of unobtrusive/unknown hand hygiene observers on hand hygiene compliance data. Hand hygiene compliance in specific patient populations and situations. The role of hand hygiene compliance during invasive procedures (eg, line insertion) as compared with its importance during non-invasive procedures (eg, abdominal ultrasound).
Physicians and hand hygiene	Impact of evaluation and feedback to improve physicians’ hand hygiene practices and sustained gains achieved. The role of physicians as champions in providing feedback on hand hygiene compliance for medical colleagues, students and other professionals.
Monitoring hand hygiene performance	Comparative evaluation of the accuracy of different monitoring techniques such as ABHR monitoring and their combined role in infectious outcomes. Impact of individual healthcare workers hand hygiene performance on healthcare workers’ group hand hygiene compliance. The role of systematic monitoring and feedback in providing healthcare workers with a risk-based learning opportunity. The role of systems to monitor microbiological events (eg, transmission of multidrug-resistant pathogens) in real time and highlight times for hand hygiene to break the transmission chain. The representativeness of hand hygiene compliance auditing during daytime care as opposed to other shifts (evenings/nights/weekends. The role of new electronic compliance monitoring approaches based on the ‘5 Moments’ to reduce auditing time and improve efficiency. The role of new electronic monitoring approaches to improve the quality (volume, duration, technique, coverage) of hand hygiene action. Frequency of auditing that should be undertaken in non-acute facilities (including sites such as dental health, mental health and primary care settings). Methods for measuring hand hygiene adherence in non-hospital settings (such as home care, ambulatory care, emergency medical services, nursing homes, long-term care, etc.). Standards to be assessed in monitoring hand hygiene compliance in settings with limited resource. The feasibility of monitoring all ‘5 Moments’ for hand hygiene in low-resource settings. The cost-effectiveness of hand hygiene monitoring apps. The acceptability of electronic monitoring devices. The cost-effectiveness of electronic monitoring devices. Effectiveness of monitoring ABHR and antimicrobial soap consumption as a surrogate for direct observation of hand hygiene compliance monitoring. Impact on individual healthcare worker hand hygiene performance of his/her sense of serving as a role model for hand hygiene. The acceptability of hand hygiene monitoring apps.
Performance feedback	Factors influencing the effectiveness of hand hygiene performance feedback. How best to apply performance feedback in hand hygiene, incorporating an underlying behavior change conceptual model and involving investigators with expertise in sociology, psychology and management science. Cost-effectiveness of different performance feedback approaches/strategies. The impact of performance feedback on hand hygiene compliance (taking numerous contexts into account, such as baseline hand hygiene compliance, simultaneous hand hygiene promotion interventions, and organizational structure). Methods to effectively implement/present performance feedback for healthcare workers’ hand hygiene improvement. Develop and test automated systems for flexible and continuous performance feedback and validation in comparison to traditional methods. Use of data on barriers and predictors of hand hygiene compliance during feedback to improve hand hygiene action. Assess the impact of individualized/personalized versus group feedback on hand hygiene compliance. The role of target setting embedded in performance feedback on hand hygiene compliance.
Monitoring your institution (Hand Hygiene Self-Assessment Framework (HHSAF))	Benchmarking variables for comparing hand hygiene compliance in facilities of different sizes and complexity. The role and cost-effectiveness of semi-automated and electronic tools to facilitate completion of and feedback from the HHSAF at the healthcare facility level. Approaches for comparing HHSAF results from healthcare facilities in countries with different socioeconomic background and resource availability.
External regulation and evaluation	The role of regional/national standards/regulations on hand hygiene practices across settings. Methods for assessing hand hygiene compliance by external evaluators such as accrediting bodies and government regulators versus internal hospital auditing.
**Communication and Reminders**	
Hand hygiene promotion	To determine the effectiveness of different elements of communication strategies focused on the importance/role of hand hygiene on the hand hygiene behavior of health workers. To assess the relationship between reminders in the workplace (eg, posters, stickers, visual and vocal prompts, banners, screensavers) and sustained hand hygiene improvement in health care. To evaluate the difference between locally and centrally developed reminders in the workplace (eg, posters, stickers, visual and vocal prompts, banners, screensavers) in terms of impact (on both the immediate and long-term hand hygiene behaviors of health workers). To evaluate the optimal strategies for involving patients in the design and implementation of hand hygiene promotional activities. To evaluate the optimal strategies for involving health workers (eg, at the ward, service, department level) in the design and implementation of hand hygiene promotional activities. To evaluate the cost-effectiveness of hand hygiene promotion strategies in different healthcare settings.
Hand hygiene marketing approaches	To evaluate the impact of social marketing approaches on health worker behavior change. To evaluate the impact of targeted marketing strategies on the knowledge and perceptions of healthcare workers from different groups and cultures (eg, professional categories, gender, undergraduate trainees). To study the relative contribution of innovative approaches used to market hand hygiene on both short- and long-term hand hygiene compliance.
Hand hygiene campaigns and communication	To identify factors at the national, sub-national and international level that influence the development and sustainability of hand hygiene campaigns. To evaluate the impact of engaging with communities in the development of global hand hygiene campaigns. To determine the influence of message framing and use of language within hand hygiene campaigns across different cultures, contexts and cadres of the health workforce (including leaders) on health workers’ hand hygiene behaviors. To evaluate and (if possible) quantify the impact of emergency events (such as H1N1 or Covid-19 pandemics) on the development and impact of hand hygiene communication strategies and campaigns.
**Institutional and Safety Climate**	
Safety climate/culture change	Influence of different cadres of the health workforce on institutional safety climate. Perspectives of different cadres of health workers towards an institutional safety climate. The relationship between a health care facility’s safety and quality climate/culture and the culture related to hand hygiene (and infection prevention and control). The influence of a health care facility’s safety and quality climate/culture on hand hygiene practices during outbreaks/emergencies/pandemics. The role of hand hygiene campaigns (including promotional messages and campaign communications, reminders in the workplace) in shaping/influencing a sustained institutional safety climate.
Personal accountability for hand hygiene	Best methods for measuring personal accountability (health workers are accountable for their hand hygiene behavior). The relationship between training of individual health workers and personal accountability for hand hygiene improvement. The influence of an enabling environment (built environment, materials and equipment for hand hygiene) on personal accountability for hand hygiene. The relationship between different methods for monitoring and feedback of hand hygiene performance and personal accountability for hand hygiene. The impact of different types of appraisal/reward systems/incentives (including financial) on personal accountability for hand hygiene. The relationship between individual health worker perceptions of hand hygiene and personal accountability. The influence of hand hygiene champions/role models (people providing the example/advocating for the causes of patient safety and hand hygiene standards) on personal accountability for hand hygiene. The relationship between a leadership approach that demonstrably values hand hygiene (eg, allocates resources, plans, evaluates, contextualizes, refreshes strategies for hand hygiene improvement) and the personal accountability of individual health workers. The factors that influence the development (eg, training, mentoring, attitudes, beliefs, values) of an effective hand hygiene champion.
Leadership	The effectiveness of a leadership approach that demonstrably values hand hygiene through a MMIS to improve the overall institutional safety climate. The most effective governance structures for shaping/influencing an institutional safety climate that supports hand hygiene. The barriers and drivers at the leadership/management and individual level to institutionalize hand hygiene as a priority. The influence of infection prevention and control/hand hygiene training targeted at hospital leadership on an institutional safety climate. The direct relationship between leadership support for hand hygiene and hand hygiene improvement/performance. The leadership factors influencing an institution’s commitment to hand hygiene improvement. The relationship between a national infection prevention and control program (according to the requirements laid out in WHO national-level core components) and its relevance and influence to an institutional safety climate.
Patient participation and empowerment	The relationship between patient participation/empowerment strategies and the establishment of an institutional safety climate that values hand hygiene. The factors that motivate decision-makers/senior managers to involve patients within institutional strategies for improving hand hygiene. The impact of patient participation/empowerment on hand hygiene improvement including the influence of patient participation on hand hygiene improvement at different implementation stages (eg, program design and point of care). The relationship between national culture characteristics (especially dimensions of power distance and uncertainty avoidance) and patient participation/empowerment in hand hygiene initiatives. The most effective methods of patient participation/empowerment to improve institutional hand hygiene practices. Barriers and facilitators of patient participation/empowerment in hand hygiene interventions. The role and impact of visitors and informal caregivers in hand hygiene improvement. The perceptions of service users and patients towards an institutional safety climate and its impact on hand hygiene standards.
Religion and tradition	The influence and impact of wider societal norms (including national and organizational culture, religion and traditions) on the institutional safety climate that influences hand hygiene.
**HAI Impact**	
Effect of hand hygiene on transmission, colonization and/or infection	To explore which hand hygiene compliance measurement (eg, direct observation, electronic monitoring, AHBR consumption or other measurements) best correlates with transmission/ colonization/infection outcomes. To establish the timespan between the implementation of the intervention aimed at hand hygiene improvement and detection of a demonstrable impact on outcomes. To establish the minimum incremental percentage improvement of hand hygiene compliance (depending on different baseline compliance levels) to achieve a significant impact on outcomes. To assess the effect of hand hygiene promotion as a single intervention (in addition to regular implementation of other best practices) on different types of HAIs (eg, caused by microorganisms of epidemiological significance such as multidrug-resistant organisms). To determine the importance of hand hygiene in preventing specific types of HAIs when using bundles and/or MMIS for the reduction of specific infections. To estimate the impact of hand hygiene promotion on HAI reduction and on lives saved (ie, mortality, quality-adjusted life years or disability-adjusted life years). To identify hand hygiene products (used appropriately according to the optimal technique) that are effective in preventing transmission of and removing *Clostridioides difficile* spores from health workers’ hands (such as hand hygiene with soap and water versus AHBR). To determine the association between hand hygiene compliance increase and reduction of transmission/colonization /infection by microorganisms of interest (including multidrug-resistant organisms) (eg, non-linear relationships: threshold effects. etc.). To assess the role of improving hand hygiene only (independent of contact precautions) for sustained control of drug-resistant organisms. To assess the effectiveness of patients’ respiratory etiquette and hand hygiene to prevent transmission of respiratory viruses, including influenza and coronaviruses. To develop clinical and experimental models to study cross-contamination from patient to patient and from the environment to patients. To assess the effectiveness of hand hygiene improvement to reduce the spread of noroviruses and other viruses/microorganisms of interest. To determine the importance of hand hygiene versus environmental hygiene versus the level of patient personal hygiene on microbial transmission – the respective and combined impact.
Hand hygiene in specific settings	To evaluate the impact of hand hygiene compliance on mortality attributable to HAI in patients admitted to the intensive care unit. To evaluate the role of hand hygiene compliance on carriage and transmission of SARS-CoV-2 and other respiratory viruses. To assess the impact of hand hygiene improvement on pathogen transmission /colonization /infection in long-term care and home care. To assess the impact of hand hygiene improvement on pathogen transmission /colonization /HAIs in ambulatory care. To assess the impact of hand hygiene improvement on pathogen transmission/ colonization/ dialysis-associated infections. To develop and test the impact of suitable strategies to achieve hand hygiene improvement and integration in the workflow of a dialysis session. To identify and assess the role of patient empowerment in achieving hand hygiene practices in hemodialysis and its impact on reducing transmission/colonization/HAI. To assess the provision of targeted education on hand hygiene for anesthesiologists on the reduction of hand contamination and infection risk during anesthesiology practices. To assess the role of anesthesiologists’ hand hygiene compliance and their direct implication in infections acquired during anesthesiology practices (eg, vascular-catheter-associated bacteremia or meningitis following spinal anesthesia). To determine the impact of improved hand hygiene compliance (both surgical hand preparation and hand hygiene practices in the surgical ward) on surgical site infection rates. To assess the effectiveness of hand hygiene improvement strategies on preventing infection acquisition in community healthcare settings (such as clinics, outpatient dialysis units, etc.).
Hand hygiene in settings with limited resources	To develop feasible standardized methods and indicators for HAI surveillance and hand hygiene compliance monitoring for both local evaluation and international benchmarking, and establish the link with HAI reduction in low-resource settings. To identify the best methods for assessing the large-scale feasibility of hand hygiene improvement strategies in low-resource settings. To identify the best methods for assessing the cost-effectiveness of hand hygiene improvement strategies in low-resource settings. To assess the impact of implementation of a hand hygiene MMIS on hand hygiene compliance and transmission/colonization/HAI rates in low-resource settings. To determine the impact of availability of resources needed for hand hygiene (supplies, staff for hand hygiene promotion/implementation, eg, IPC nurses) on hand hygiene compliance and transmission/colonization/HAI rates. To explore the impact of hand hygiene compliance on infectious outcomes in settings where laboratory resources are scarce and syndromic detection of infection is used as a surrogate for laboratory-based detection.
The economic impact of improved hand hygiene	To assess the cost-effectiveness of MMIS. To develop mathematical models to explore the cost-effectiveness of different approaches to hand hygiene improvement. To explore how financial resources dedicated to hand hygiene evolve as hand hygiene improvement strategies mature and become routine in health services.

AHBR, alcohol-based hand rub; MMIS, multimodal improvement strategy. Research priorities were identified and ranked based on the Delphi process conducted with a panel of international experts.

**Table 3:** Characteristics of Delphi panel of international experts

	Institutional Safety Climate n = 48 (%)	Evaluation and Feedback n = 45 (%)	System Change n = 33 (%)	Training and Education n = 48 (%)	Communication and Reminders n = 37 (%)	HAI Impact n = 43 (%)
**WHO region**						
Africa	6 (12.5)	3 (6.7)	3 (9.1)	2 (4.2)	2 (5.4)	5 (11.6)
Americas	12 (25.0)	7 (15.6)	6 (18.2)	9 (18.8)	8 (21.6)	6 (14.0)
Eastern Mediterranean	3 (6.2)	4 (8.9)	0 (0)	3 (6.2)	0 (0)	4 (9.3)
Europe	11 (22.9)	7 (15.6)	7 (21.2)	9 (18.8)	10 (27.0)	4 (9.3)
South-East Asia	10 (20.8)	8 (17.8)	6 (18.2)	4 (8.3)	3 (8.1)	7 (16.3)
Western Pacific	6 (12.5)	5 (11.1)	3 (9.1)	5 (10.4)	5 (13.5)	3 (7.0)
**World Bank income level**						
High	28 (58.3)	19 (42.2)	15 (45.5)	21 (43.8)	19 (51.4)	14 (32.6)
Upper-middle-income	9 (18.8)	4 (8.9)	3 (9.1)	4 (8.3)	4 (10.8)	5 (11.6)
Lower-middle-income	6 (12.5)	9 (20.0)	4 (12.1)	5 (10.4)	3 (8.1)	6 (14.0)
Low-income	5 (10.4)	2 (4.4)	3 (9.1)	2 (4.2)	2 (5.4)	4 (9.3)
**Area of expertise**						
Infection prevention and control	40 (83.3)	43 (95.6)	31 (93.9)	45 (93.8)	33 (89.2)	41 (95.3)
Hand hygiene	33 (68.8)	43 (95.6)	32 (97.0)	40 (83.3)	30 (81.1)	43 (100.0)
Infectious disease	24 (50.0)	36 (80.0)	23 (69.7)	30 (62.5)	23 (62.2)	21 (48.8)
Microbiology	15 (31.2)	23 (51.1)	17 (51.5)	12 (25.0)	9 (24.3)	29 (67.4)
Patient safety quality	27 (56.2)	28 (62.2)	19 (57.6)	22 (45.8)	13 (35.1)	23 (53.5)
Academic research	25 (52.1)	34 (75.6)	28 (84.8)	33 (68.8)	24 (64.9)	35 (81.4)
Guideline development	22 (45.8)	37 (82.2)	29 (87.9)	28 (58.3)	21 (56.8)	37 (86.0)
Water, sanitation and hygiene	6 (12.5)	21 (46.7)	14 (42.4)	7 (14.6)	6 (16.2)	16 (37.2)
Other	8 (16.7)	26 (57.8)	16 (48.5)	0 (0.0)	0 (0.0)	21 (48.8)
**Years of experience in the current expertise**						
5-10	8 (16.7)	3 (6.7)	2 (6.1)	4 (8.3)	2 (5.4)	1 (2.3)
10-15	7 (14.6)	12 (26.7)	8 (24.2)	13 (27.1)	9 (24.3)	9 (20.9)
>15	21 (43.8)	21 (46.7)	0 (0)	31 (64.6)	26 (70.3)	33 (76.7)
-	12 (25.0)	9 (20.0)	0 (0)	0 (0)	0 (0)	0 (0)

**Table 4:** Highest hand hygiene research priorities categorized into six thematic domains following expert panel consensus

	Agree, %	Neutral, %	Disagree, %	Unable to rate, %	Mean +- SD
**System change**					
The approaches or interventions* required to facilitate sustained system change in the context of the multimodal improvement strategy (MMIS).	97.0	3.0	0.0	0.0	1.38 +/- 0.54
Hand hygiene agents’ efficacy in removing a range of organisms from health worker hands, including *C. difficile* spores and respiratory viruses, and the impact on transmission and HAI.	95.0	6.0	0.0	0.0	1.33 +/- 0.58
The use of gloves and the influence on hand hygiene adherence and pathogen transmission.	97.0	0.0	3.0	0.0	1.3 +/- 0.62
**Training and Education**					
To evaluate the impact of different hand hygiene training and educational strategies (face-to-face and virtual, participatory, team and task-based strategies that are participatory and include bedside and simulation) on the knowledge and skills (eg, appropriateness of hand hygiene technique) of health workers across the levels of the health system (primary, secondary, tertiary and long- term care).	98.0	3.0	0.0	0.0	1.23 +/- 0.49
To identify the optimal educational methods to improve health worker understanding of the dynamics of transmission at the point of (patient) care.	97.0	3.0	0.0	0.0	1.42 +/- 0.55
To determine the effectiveness of the train-the-trainer approach on sustained hand hygiene improvement across settings and populations.	95.0	6.0	0.0	0.0	1.5 +/- 0.61
**Evaluation and Feedback**					
Use of data on barriers and predictors of hand hygiene compliance during feedback to improve hand hygiene action.	94.0	6.0	0.0	0.0	1.37 +/- 0.59
Impact of evaluation and feedback to improve physicians’ hand hygiene practices, and sustain gains achieved.	96.0	4.0	0.0	0.0	1.41 +/- 0.57
The impact of performance feedback on hand hygiene compliance (taking numerous contexts into account, such as baseline HH compliance, simultaneous hand hygiene promotion interventions, and organizational structure).	92.0	8.0	0.0	0.0	1.58 +/- 0.64
**Communication and Reminders**					
To determine the effectiveness of different elements of communication strategies focused on the importance/role of hand hygiene on hand hygiene behavior of health workers.	94.0	3.0	0.0	3.0	1.46 +/- 0.56
To evaluate the cost-effectiveness of hand hygiene promotion strategies in different health care settings.	93.0	5.0	3.0	0.0	1.5 +/- 0.73
To determine the influence of message framing and use of language within hand hygiene campaigns across different cultures, contexts and cadres of the health workforce (including leaders) on health workers’ hand hygiene behaviors.	92.0	5.0	0.0	3.0	1.48 +/- 0.59
**Institutional and Safety Climate**					
The relationship between a health care facility’s safety and quality climate/culture and the culture related to hand hygiene (and infection prevention and control).	90.0	6.0	2.0	2.0	1.51 +/- 0.7
Influence of different cadres of the health workforce on institutional safety climate.	86.0	10.0	4.0	0.0	1.72 +/- 0.8
The relationship between a leadership approach that demonstrably values hand hygiene (eg, allocates resources, plans, evaluates, contextualises, refreshes strategies for hand hygiene improvement) and the personal accountability of individual health workers.	88.0	8.0	2.0	2.0	1.59 +/- 0.73
**HAI Impact**					
To determine the association between hand hygiene compliance increase and reduction of transmission/colonization/infection by microorganisms of interest (including multidrug-resistant organisms, eg, non-linear relationships: threshold effects etc.).	96.0	2.0	2.0	0.0	1.6 +/- 0.63
To assess the impact of hand hygiene improvement on pathogen transmission/colonization/infection in long- term care and home care.	96.0	0.0	4.0	0.0	1.58 +/- 0.7
To develop feasible standardised methods and indicators for HAI surveillance and hand hygiene compliance monitoring for both local evaluation and international benchmarking, and establish the link with HAI reduction in low-resource settings.	98.0	2.0	0.0	0.0	1.32 +/- 0.51

HAI, healthcare-associated infection; AMR, antimicrobial resistance; MMIS, multimodal improvement strategy. The table presents the top three research priorities from each of the six thematic domains, based on the highest consensus agreement achieved by the expert panel and the highest mean score.
