# Supplementary material for: WHO Global Research Agenda for Hand Hygiene Improvement in Health Care: a Delphi Consensus Study
Source: Infect Control Hosp Epidemiol. Author manuscript; Available in PMC 2025 May 1. (PMC7617569; doi:10.1017/ice.2025.32)
Supplement: Supplemental File [file EMS204160-supplement-Supplemental_File.pdf]

Supplemental File

Appendix to: Allegranzi et al. **WHO Global Research Agenda for Hand Hygiene Improvement in Health Care: a Delphi Consensus Study**

Contents

1. **Appendix A.** Expert-proposed research statements on hand hygiene in healthcare.
2. **Appendix B.** Results of round 1 Delphi surveys on the 2023-2030 Hand Hygiene Research Agenda.
3. **Appendix C.** Round 2 Delphi survey results: 2023-2030 Hand Hygiene Research Agenda, including non-consensus statements ……………………………………………………………………………………………….

Appendix A. Expert-proposed research statements on hand hygiene in healthcare.

Table 1. Expert-proposed research statements on hand hygiene in healthcare: categorized by thematic domains.

Thematic Domain	Subdomain	Research statement
**System Change**	General system change – enabling environment	To assess the role of public health emergencies of national/international concern on availability of hand hygiene infrastructure, materials and equipment and the impact on hand hygiene compliance.
	Hand hygiene agents	Economic evaluation of local alcohol-based handrub (ABHR) production versus local market availability.
	The skin microbiome	The extent of colonization from healthcare worker hand microbial flora and the correlation with HAI in pre-determined hospitalized patients.
	Dynamics of hand transmission	The minimum infectious dose of potentially pathogenic organisms on environmental contamination and the impact on the times for hand hygiene.
		The role of the built environment and ergonomics/human factors in hand recontamination.

**Evaluation and Feedback**	Monitoring hand hygiene performance	To assess the cost-effectiveness and acceptability of hand hygiene monitoring applications and electronic monitoring devices.
	External regulation and evaluation	The role of regional/national standards/regulations on hand hygiene practices across settings.
**Communication and Reminders**	Hand hygiene campaigns and communication	To evaluate and (if possible) quantify the impact of emergency events (such as H1N1 or Covid-19 pandemics) on the development and impact of hand hygiene communication strategies and campaigns.
**Institutional and Safety Climate**	Patient participation and empowerment	The relationship between national culture characteristics (especially dimensions of power distance and uncertainty avoidance) and patient participation/empowerment in hand hygiene initiatives.
**HAI Impact**	Hand hygiene in specific settings	To evaluate the role of hand hygiene compliance on carriage and transmission of SARS-CoV-2 and other respiratory viruses.
	Hand hygiene in settings with limited resources	To assess the impact of implementation of a hand

		hygiene MMIS on hand hygiene compliance and transmission/colonization/HAI rates in low-resource settings.
		To determine the impact of availability of resources needed for hand hygiene (supplies, staff for hand hygiene promotion/implementation, eg, IPC nurses) on hand hygiene compliance and transmission/colonization/HAI rates.

Appendix B. Results of round 1 Delphi surveys on the 2023-2030 Hand Hygiene Research Agenda.

Table 2. Results of round 1 Delphi surveys

Domain1. Safety climate

	Agree (%)	Neutral (%)	Disagree (%)	Unable to rate (%)	Mean ± SD
**Safety climate/culture change**					
Influence of different cadres of the health workforce on institutional safety climate	86	10	4	0	1.72 +/-0.8
Perspectives of different cadres of health workers towards an institutional safety climate	84	14	2	0	1.86 +/- 0.72
The relationship between a healthcare facility's safety and quality climate/culture and the culture related to hand hygiene (and infection prevention and control)	90	6	2	2	1.51 +/-0.7
The influence of a health care facility's safety and quality climate/culture on hand hygiene practices during outbreaks/emergencies/pandemics	82	14	4	0	1.76 +/- 0.84
The role of media (mainstream and social media) in shaping/influencing an institutional safety climate and hand hygiene improvement	68	24	8	0	2.14 +/- 0.96

The role of hand hygiene campaigns (including promotional messages and campaign communications, reminders in the workplace) in shaping/influencing a sustained institutional safety climate	86	8	6	0	1.74 +/- 0.91
**Personal accountability for hand hygiene**					
Best methods for measuring personal accountability (health workers are accountable for their hand hygiene behavior)	84	10	6	0	1.72 +/- 0.94
The relationship between training of individual health workers and personal accountability for hand hygiene improvement	72	16	10	2	1.96 +/- 1.05
The influence of an enabling environment (built environment, materials and equipment for hand hygiene) on personal accountability for hand hygiene	86	12	0	2	1.59 +/-0.7
The relationship between different methods for monitoring and feedback of hand hygiene performance and personal accountability for hand hygiene	84	12	2	2	1.57 +/- 0.78
The impact of different types of appraisal/reward systems/incentives (including financial) on personal accountability for hand hygiene	80	12	6	2	1.8 +/- 0.88
The relationship between individual health worker perceptions of hand hygiene and personal accountability	72	22	6	0	2.06 +/- 0.86
The influence of hand hygiene champions/role models (people providing the example/advocating for the causes of	74	18	6	2	1.92 +/- 0.97

patient safety and hand hygiene standards) on personal accountability for hand hygiene					
The factors that influence the development (e.g., training, mentoring, attitudes, beliefs, values) of an effective hand hygiene champion	66	26	6	2	2.0 +/- 0.95
The relationship between a leadership approach that demonstrably values hand hygiene (e.g., allocates resources, plans, evaluates, contextualizes, refreshes strategies for hand hygiene improvement) and the personal accountability of individual health workers	88	8	2	2	1.59 +/- 0.73
The influence of different institutional social networks on personal accountability for hand hygiene	60	34	6	0	2.24 +/- 0.93
**Leadership**					
The effectiveness of a leadership approach that demonstrably values hand hygiene through a multimodal improvement strategy to improve the overall institutional safety climate	82	12	4	2	1.65 +/- 0.85
The most effective governance structures for shaping/influencing an institutional safety climate that supports hand hygiene	86	10	0	4	1.58 +/- 0.67
The barriers and drivers at the leadership/management and individual level to institutionalize hand hygiene as a priority	86	10	4	0	1.6 +/- 0.82
The influence of infection prevention and control/hand hygiene training targeted at hospital leadership on an institutional safety climate	82	12	6	0	1.76 +/- 0.88

The direct relationship between leadership support for hand hygiene and hand hygiene improvement/performance	82	14	2	2	1.69 +/- 0.79
The leadership factors influencing an institution's commitment to hand hygiene improvement	82	14	4	0	1.74 +/- 0.84
The relationship between a national IPC program (according to the requirements laid out in WHO national level core components) and its relevance and influence to an institutional safety climate	76	20	4	0	1.88 +/- 0.93
**Patient participation and empowerment**					
The relationship between patient participation/empowerment strategies and the establishment of an institutional safety climate that values hand hygiene	86	8	6	0	1.72 +/- 0.92
The factors that motivate decision-makers/senior managers to involve patients within institutional strategies for improving hand hygiene	70	24	6	0	1.98 +/- 0.93
The impact of patient participation/empowerment on hand hygiene improvement including the influence of patient participation on hand hygiene improvement at different implementation stages (e.g., program design and point of care)	80	18	2	0	1.84 +/- 0.86
The role and impact of visitors and informal caregivers in hand hygiene improvement	65	29	6	0	2.19 +/- 0.91

The perceptions of service users and patients towards an institutional safety climate and its impact on hand hygiene standards	68	20	10	2	2.15 +/- 0.98
The relationship between national culture characteristics (especially dimensions of power distance and uncertainty avoidance) and patient participation/empowerment in hand hygiene initiatives	70	26	2	2	2.02 +/-0.8
The most effective methods of patient participation/empowerment to improve institutional hand hygiene practices	84	12	4	0	1.62 +/- 0.91
Barriers and facilitators of patient participation/empowerment in hand hygiene interventions	80	14	6	0	1.82 +/- 0.95
The underlying ethical considerations in the development of a patient participation/empowerment strategy as part of an overall approach to hand hygiene improvement	58	24	12	6	2.23 +/- 1.17
**Religion and tradition**					
The influence of different religions and traditions on hand hygiene improvement strategies, including as barriers and facilitators	68	22	10	0	2.26 +/- 0.91
The relationship between religious beliefs, practices and traditions and institutional safety climate that influences hand hygiene	60	28	10	2	2.29 +/-0.9
The influence and impact of wider societal norms (including national and organizational culture, religion and traditions)	78	18	0	4	1.83 +/- 0.72

on the institutional safety climate that influences hand hygiene					
The influence of religion and traditions on acceptance and use of alcohol-based hand hygiene products	52	24	22	2	2.55 +/- 1.21

Domain 2: Evaluation and feedback

	Agree (%)	Neutral (%)	Disagree (%)	Unable to rate (%)	Mean ± SD
**Compliance with hand hygiene best practices**					
Influence of specific cultures and social and religious environments on hand hygiene compliance by different healthcare workers	79	8	10	2	2.03 +/-1.0
Use of data on barriers and predictors of hand hygiene compliance during feedback to improve hand hygiene action	94	6	0	0	1.37 +/- 0.59
Caregivers' hand hygiene compliance in during care provided in the community (including home care)	80	8	8	4	1.85 +/- 0.96
Caregivers' hand hygiene compliance in during traditional medicine interventions	71	10	10	8	2.03 +/- 0.92

Caregivers' hand hygiene compliance during care in acute healthcare facilities	90	4	6	0	1.49 +/- 0.83
The role of unobtrusive/unknown hand hygiene observers on hand hygiene compliance data	73	19	6	2	1.83 +/- 0.95
Hand hygiene compliance in specific patient populations and situations	83	10	4	2	1.67 +/- 0.82
The role of hand hygiene compliance during invasive procedures (e.g., line insertion) as compared with its importance during non-invasive procedures (e.g., abdominal ultrasound)	82	8	8	2	1.62 +/- 1.01
**Physicians and hand hygiene**					
Impact of evaluation and feedback to improve physicians' hand hygiene practices and sustained gains achieved	96	4	0	0	1.41 +/- 0.57
The role of physicians as champions in providing feedback on hand hygiene compliance for medical colleagues, students and other professionals	85	8	4	2	1.55 +/- 0.81
**Monitoring hand hygiene performance**					
Comparative evaluation of the accuracy of different monitoring techniques such as alcohol-based hand rub (ABHR) consumption/soap consumption, direct observation and electronic monitoring and their combined role in infectious outcomes	90	6	2	2	1.46 +/-0.7

Impact of individual healthcare worker hand hygiene performance on group healthcare worker hand hygiene compliance	77	6	12	4	2.03 +/- 0.99
Impact on individual healthcare worker hand hygiene performance of his/her sense of serving as a role model for hand hygiene	63	23	13	2	2.26 +/- 0.96
The role of systematic monitoring and feedback in providing healthcare workers with a risk-based learning opportunity	88	10	0	2	1.65 +/- 0.66
The role of systems to monitor microbiological events (e.g., transmission of multiresistant pathogens) in real time and to highlight times for hand hygiene to break the transmission chain	81	6	10	2	1.79 +/- 1.07
The representativeness of hand hygiene compliance auditing during daytime care as opposed to other shifts (evenings/nights/weekends)	79	8	12	0	1.99 +/- 1.01
The role of new electronic compliance monitoring approaches based on the 5 Moments to reduce auditing time and improve efficiency	82	8	4	6	1.72 +/- 0.87
The role of new electronic monitoring approaches to improve the quality (volume, duration, technique, coverage) of hand hygiene action	87	8	0	4	1.54 +/- 0.65
Frequency of auditing that should be undertaken in non-acute facilities (including sites such as dental health, mental health and primary care settings)	81	13	4	2	1.98 +/- 0.73

Methods for measuring hand hygiene adherence in non-hospital settings (such as home care, ambulatory care, emergency medical services, nursing homes, long-term care, etc.)	92	6	0	2	1.67 +/- 0.59
Standards to be assessed in monitoring hand hygiene compliance in settings with limited resource	90	4	4	2	1.46 +/- 0.76
The feasibility of monitoring all 5 Moments for hand hygiene in low-resource settings	77	10	8	4	1.75 +/- 1.02
The acceptability of hand hygiene monitoring apps	67	25	6	2	2.18 +/- 0.88
The cost-effectiveness of hand hygiene monitoring apps	73	19	6	2	2.14 +/- 0.77
The acceptability of electronic monitoring devices	73	15	10	2	2.16 +/- 0.99
The cost-effectiveness of electronic monitoring devices	82	10	6	2	1.84 +/- 0.91
Effectiveness of monitoring ABHR alcohol-based hand rub and antimicrobial soap consumption as a surrogate for direct observation of hand hygiene compliance monitoring	70	13	10	6	2.0 +/- 1.03

Performance feedback

Factors influencing the effectiveness of hand hygiene performance feedback	92	8	0	0	1.56 +/- 0.64
How best to apply performance feedback in hand hygiene, incorporating an underlying behavioral change conceptual model and involving investigators with expertise in sociology, psychology and management science	90	8	2	0	1.43 +/- 0.72
Cost-effectiveness of different performance feedback approaches/strategies	90	10	0	0	1.58 +/- 0.67
The impact of performance feedback on hand hygiene compliance (taking numerous contexts into account, such as baseline hand hygiene compliance, simultaneous hand hygiene promotion interventions, and organizational structure)	92	8	0	0	1.58 +/- 0.64
Methods to effectively implement/present performance feedback for healthcare workers' hand hygiene improvement	85	10	4	0	1.64 +/- 0.82
Develop and test automated systems for flexible and continuous performance feedback and validation in comparison to traditional methods	81	15	4	0	1.9 +/- 0.79
Use of data on barriers and predictors of hand hygiene compliance during feedback to improve hand hygiene action	82	13	4	2	1.81 +/- 0.88
Assess the impact of individualized/personalized versus group feedback on hand hygiene compliance	77	21	2	0	1.94 +/- 0.85

The role of target setting embedded in performance feedback on hand hygiene compliance	81	10	4	4	1.88 +/- 0.83
**Monitoring your institution (Hand Hygiene Self-Assessment Framework [HHSAF])**					
Benchmarking variables for comparing hand hygiene compliance in facilities of different sizes and complexity	79	10	8	2	1.79 +/- 0.93
Approaches for comparing HHSAF results from healthcare facilities in countries with different socioeconomic background and resource availability	69	21	10	0	2.12 +/-1.0
The role and cost-effectiveness of semi-automated and electronic tools to facilitate the completion of and feedback from the HHSAF at the healthcare facility level	71	23	4	2	2.08 +/- 0.87
**External regulation and evaluation**					
The role of regional/national standards/regulation on hand hygiene practices across settings	84	15	2	0	1.73 +/- 0.78
Methods for assessing hand hygiene compliance by external evaluators such as accrediting bodies and government regulators versus internal hospital auditing	85	10	4	0	1.7 +/- 0.88

Domain 3: System change

	Agree (%)	Neutral (%)	Disagree (%)	Unable to rate (%)	Mean ± SD
**General system change – enabling environment**					
The approaches or interventions required to facilitate sustained system change in the context of a multimodal improvement strategy	97	3	0	0	1.38 +/- 0.54
The role of legislation/policy and mechanisms for enforcement on sustained system change and the impact on hand hygiene compliance	91	3	6	0	1.65 +/- 0.91
The prevailing unintended consequences on the healthcare system transpiring from sustained system change efforts in the context of the multimodal improvement strategy	70	18	a	0	2.13 +/- 1.06
The factors in the enabling environment that determine hand hygiene station availability, quantity, distribution and location	85	12	3	0	1.62 +/- 0.81
The relationship between hygiene service ladder levels, as defined by the United States Joint Monitoring Program (basic, limited and no services) and hand hygiene compliance	73	18	6	3	2.04 +/- 0.84
The relationship between hygiene service ladder levels as defined by the Joint Monitoring Program (basic, limited, and no services) and healthcare-associated infection (HAI)	73	15	6	6	2.01 +/- 0.83
The role of climate change and other public health emergencies of national/international concern on	67	15	15	3	2.16 +/- 1.02

availability of hand hygiene infrastructure, materials and equipment and the impact on hand hygiene compliance					
Accessibility policies/standards for health workers, patients (including children) and visitors that address equity, diversity and inclusivity, and the relationship with hand hygiene compliance	76	15	6	3	2.04 +/- 0.91
**Surgical hand preparation**					
The optimal hand hygiene technique for surgical hand preparation to ensure improved antimicrobial activity as well as tolerability to agents	94	0	0	6	1.5 +/-0.5
The role of powdered gloves and their effect on the quality of surgical hand preparation	76	12	6	6	1.91 +/- 0.85
The availability of potable/drinking water and its impact on compliance with surgical hand preparation	76	6	9	9	1.95 +/- 0.99
The role of hand hygiene agents' tolerability and acceptability on surgeons' skin and the relationship with adherence to surgical hand preparation	94	0	0	6	1.47 +/-0.5
Alcohol-based surgical hand preparation compared to traditional hand scrubbing and the implications for microbial load, water consumption and waste production	85	3	6	6	1.56 +/- 0.94
Determining flash points for flammability of ABHR in relation to efficacious concentrations for hand hygiene	41	26	27	6	2.79 +/- 1.03
**Hand hygiene agents**					
Economic evaluation of local ABHR production versus local market availability	85	12	3	0	1.86 +/- 0.85

The factors that facilitate availability of locally-produced, efficacious and cost-effective hand hygiene agents	88	9	3	0	1.74 +/- 0.86
The development of international standards and norms on assessing the antimicrobial activity of hand hygiene agents	85	15	0	0	1.71 +/- 0.71
The development of (new) international standards and norms and their influence on agent availability in low- and middle-income countries	79	9	12	0	1.98 +/- 1.05
Hand hygiene agents' efficacy in removing a range of organisms from health worker hands, including *Clostridioides difficile* spores and respiratory viruses, and the impact on transmission and HAI	95	6	0	0	1.33 +/- 0.58
The role of emollients and agents in gel or foam form on the efficacy, tolerability and acceptability of ABHR formulations	88	6	3	3	1.76 +/-0.7
The predictive model (including concentration and purity) that can be used in the formulation of new effective, acceptable and tolerable hand hygiene agents	70	18	9	3	2.1 +/-0.9
The impact of refilling bottles with ABHR on the efficacy of the agent	73	12	12	3	2.01 +/- 1.08
**Reactions to hand hygiene agents**					
The impact of dermal harm from hand hygiene agents in a range of settings and skin types (as reported to occupational health) and the relationship with hand hygiene compliance	88	3	6	3	1.82 +/- 0.76

The impact of dermal harm from hand hygiene agents in a range of settings and skin types (as reported to occupational health) and the relationship with healthcare worker work	70	12	15	3	2.1 +/- 1.03
The role of dermal and respiratory absorption rates of alcohol during the use of ABHR and the correlation with harms (acceptable level of absorption/inhalation)	45	29	21	6	2.56 +/- 1.12
**Barriers/enablers to hand hygiene compliance**					
The use of gloves and the influence on hand hygiene adherence and pathogen transmission	97	0	3	0	1.3 +/- 0.62
The correlation between workload: staffing ratio, the built environment and hand hygiene compliance including during outbreaks	94	0	6	0	1.47 +/- 0.78
The factors that influence the selection of ABHR (e.g., ambient environment/temperature and humidity on drying time, ease of spread on hands, and stickiness of different hand rubs/staff comfort, cost) and the correlation with hand hygiene compliance	73	15	12	0	2.1 +/- 0.95
The factors that influence the use of ABHR and the relationship with hand hygiene compliance	79	9	9	3	1.86 +/- 0.93
Hand drying options and the impact on organism removal and hand hygiene compliance	80	9	12	0	1.74 +/- 1.04
The location of hand hygiene facilities, both sink and ABHR placement (agent: bed ratio) and the relationship with hand hygiene compliance	94	0	6	0	1.65 +/- 0.88

The role of sink placement and water splashes and the relationship with hand hygiene adherence and HAI in intensive care units, neonatal units	82	12	3	3	1.7 +/-0.8
The role of automated dispensers, their placement and their influence on hand hygiene compliance	79	12	9	0	1.89 +/- 0.94
The role of human factors on hand hygiene compliance	82	12	6	0	1.77 +/- 0.88
**The skin microbiome**					
Development of sample collection and testing methods to understand the dynamics of hand microbiota	70	9	18	3	2.1 +/- 1.17
Methods for determining hand contamination from a pre-determined list of touch points in clinical settings	67	18	12	3	2.07 +/- 1.11
Effective (including cost-effective) methods to culture laboratory-adapted as well as field strains of gastrointestinal and respiratory viruses	64	18	12	6	2.04 +/- 1.05
The relationship between the quantity of gastrointestinal and respiratory viruses present in the environment and the need for hand hygiene action to interrupt environmental contamination and transmission	70	9	18	3	2.16 +/- 1.22
The role of hand hygiene agents and moisturizers on the skin microbiome/health	85	6	6	3	1.73 +/- 0.83
The role of hand microbiota and the impact on infection acquisition in a range of commonly encountered interventions, eg, long lines, wounds, etc	76	9	3	12	1.74 +/- 0.78

The role of commensal hand microbial flora in resisting colonization by commonly known healthcare pathogens	73	12	9	6	1.95 +/- 1.04
The extent of colonization from healthcare worker hand microbial flora and the correlation with HAI in pre-determined hospitalized patients	79	9	6	6	1.82 +/- 1.05
The role of the skin microbiome across different age groups and the correlation with microorganism transmission and HAI	74	12	9	6	2.09 +/- 0.95
The role of 'biotics' in supporting healthy hand microbiome flora	50	32	9	9	2.42 +/- 0.84
The minimum level of potentially pathogenic organisms that need to be present on hands to cause transmission and HAI in a range of settings	76	9	12	3	1.92 +/-1.0
**Dynamics of hand transmission**					
The role of fomite contamination in hand contamination with different microbes, the potential for transmission and correlation with HAI	91	6	0	3	1.67 +/- 0.59
The minimum infectious dose of potentially pathogenic organisms on environmental contamination and the impact on the times for hand hygiene	73	15	12	0	2.1 +/- 1.04
The type of hand-to-surface touch, which occurs when different potentially pathogenic organisms are present and the influence on hand hygiene opportunities	50	29	18	3	2.48 +/-1.0
The role of the built environment and ergonomics/human factors in hand recontamination	88	9	3	0	1.86 +/- 0.69

The impact of glove removal on hand (re)contamination from pathogenic organisms in a range of settings	82	12	0	6	1.66 +/- 0.69

Domain 4: Communication and reminders

	Agree (%)	Neutral (%)	Disagree (%)	Unable to rate (%)	Mean ± SD
**Hand hygiene promotion**					
To determine the effectiveness of different elements of communication strategies focused on the importance/role of hand hygiene on hand hygiene behavior of health workers	94	3	0	3	1.46 +/-0.56
To assess the relationship between reminders in the workplace (e.g., posters, stickers, visual and vocal prompts, banners, screensavers) and sustained hand hygiene improvement in health care	79	16	5	0	1.73 +/-0.9
To evaluate the difference between locally and centrally developed reminders in the workplace (e.g., posters, stickers, visual and vocal prompts, banners, screensavers) in terms of impact (on both immediate and long-term hand hygiene behaviors of health workers)	71	24	3	3	1.91 +/-0.87

To evaluate the optimal strategies for involving patients in the design and implementation of hand hygiene promotional activities	71	26	3	0	1.95 +/-0.86
To evaluate the optimal strategies for involving health workers (e.g., at the ward, service, department level) in the design and implementation of hand hygiene promotional activities	87	11	3	0	1.59 +/-0.8
To evaluate the cost-effectiveness of hand hygiene promotion strategies in different healthcare settings	93	5	3	0	1.5 +/-0.73
**Hand hygiene marketing approaches**					
To evaluate the impact of social marketing approaches on health worker behavior change	78	18	3	0	1.85 +/-0.82
To evaluate the impact of targeted marketing strategies on the knowledge and perceptions of healthcare workers from different groups and cultures (e.g., professional categories, gender, undergraduate trainees)	85	13	3	0	1.66 +/-0.81
To study the relative contribution of each component of the marketing mix (product, price, people, place) to effective hand hygiene marketing (i.e., not solely "promotions")	53	29	11	8	2.32 +/-0.95
To study the relative contribution of innovative approaches used to market hand hygiene on both short- and long-term hand hygiene compliance	82	5	11	3	1.77 +/-0.98
**Hand hygiene campaigns and communication**					

To identify factors at the national, sub-national and international level that influence the development and sustainability of hand hygiene campaigns	89	11	0	0	1.48 +/-0.69
To evaluate the impact of engaging with communities in the development of global hand hygiene campaigns	81	16	3	0	1.67 +/-0.85
To determine the influence of message framing and use of language within hand hygiene campaigns across different cultures, contexts and cadres of the health workforce (including leaders) on health workers' hand hygiene behaviors	92	5	0	3	1.48 +/-0.59
To evaluate and (if possible) quantify the impact of emergency events (such as H1N1 or Covid-19 pandemics) on the development and impact of hand hygiene communication strategies and campaigns	82	13	5	0	1.78 +/-0.86

Domain 4: Education and training

	Agree (%)	Neutral (%)	Disagree (%)	Unable to rate (%)	Mean ± SD
**Training and education strategies**					
To identify the optimal educational methods to improve health worker understanding of the dynamics of transmission at the point of (patient) care	97	3	0	0	1.42 +/- 0.55

To evaluate the impact of different hand hygiene training and educational strategies (face-to-face and virtual, participatory, team and task-based strategies that are participatory and include bedside and simulation) on the knowledge and skills (e.g., appropriateness of hand hygiene technique) of health workers across the levels of the health system (primary, secondary, tertiary and long-term care)	98	3	0	0	1.23 +/- 0.49
To determine the effectiveness of the train-the-trainer approach on sustained hand hygiene improvement across settings and populations	95	6	0	0	1.5 +/- 0.61
To evaluate the impact of hand hygiene training strategies that use apps, technology, gamification, smart phones, simulation, role play, or the use of audiovisual, practical training on sustained hand hygiene compliance improvement across different settings and countries	92	8	0	0	1.52 +/- 0.64
To determine the influence of different training strategies on appropriate glove use and the performance of hand hygiene at the right moment by healthcare worker category	89	11	0	0	1.61 +/- 0.68
**Hand hygiene in specific patient populations and situations**					
To determine the best approaches for training and educating health workers in specialized settings (included but not limited to intensive care, anesthesiology, surgical departments, dialysis, long-term care, ambulatory care, neonatal/pediatric)	92	6	0	3	1.38 +/-0.6
To evaluate optimal methods to train and educate health workers to become champions and role models for hand hygiene in the context of their area of specialty	91	6	3	0	1.65 +/- 0.73

To identify the best approaches to educate patients and family members in hand hygiene improvement efforts while taking social and cultural context into account	88	11	0	0	1.67 +/- 0.67
To evaluate the best approaches for training and education in resource-poor settings	95	6	0	0	1.43 +/-0.6
To determine the optimal strategy to provide hand hygiene training and education in local language while minimizing translation biases	84	11	3	3	1.74 +/- 0.77
**Training and education of infection prevention and control specialists, healthcare workers and other personnel**					
To assess the impact of in-service training on hand hygiene on health worker behavior (hand hygiene compliance) in the short- and long-term	87	6	8	0	1.66 +/-0.9
To evaluate the optimal training and education approach for health workers undertaking hand hygiene monitoring	92	8	0	0	1.58 +/- 0.64
To assess the effectiveness of a train-the-trainer approach in hand hygiene training and education strategies on the prevention of device-associated infections	78	14	6	3	1.72 +/- 0.92
To assess the effectiveness of a train-the-trainer in hand hygiene training and education strategies on the incidence of drug-resistant organisms cross-transmission in healthcare facilities	84	8	6	3	1.63 +/- 0.87
To evaluate the impact of education strategies on hand hygiene knowledge across different audiences (e.g., managers versus healthcare workers)	74	23	0	3	1.82 +/- 0.79

Domain 5.HAI impact

	Agree (%)	Neutral (%)	Disagree (%)	Unable to rate (%)	Mean ± SD
**Effect of hand hygiene on transmission, colonization and/or infection**					
To explore which hand hygiene compliance measurement (e.g., direct observation, electronic monitoring, AHBR consumption or other measurements) best correlates with transmission/colonization/infection outcomes	91	2	4	2	1.55 +/- 0.73
To establish the timespan between the implementation of the intervention aimed at hand hygiene improvement and detection of a demonstrable impact on outcomes	81	11	9	0	1.98 +/- 0.94
To establish the minimum incremental percentage improvement of hand hygiene compliance (depending on different baseline compliance levels) to achieve a significant impact on outcomes	86	11	2	0	1.71 +/- 0.74
To assess the effect of hand hygiene promotion as a single intervention (in addition to regular implementation of other best practices) on different types of HAI (e.g., caused by microorganisms of epidemiological significance, such as multidrug-resistant organisms)	87	9	4	0	1.69 +/-0.8

To determine the importance of hand hygiene in preventing specific types of HAIs when using bundles and/or multimodal strategies for the reduction of specific infections	82	9	7	2	1.82 +/- 0.87
To estimate the impact of hand hygiene promotion on HAI reduction and on lives saved (i.e., mortality, quality-adjusted life years or disability-adjusted life years)	84	11	4	0	1.65 +/- 0.83
To identify hand hygiene products (used appropriately according to the optimal technique) that are effective in preventing transmission of and removing *C. difficile* spores from health workers' hands (such as hand hygiene with soap and water versus AHBR)	80	16	2	2	1.78 +/- 0.79
To determine the association between hand hygiene compliance increase and reduction of transmission/colonization/infection by microorganisms of interest (including multidrug-resistant organisms) (e.g., non-linear relationships: threshold effects, etc.)	96	2	2	0	1.6 +/- 0.63
To assess the role of improving hand hygiene only (independent of contact precautions) for the sustained control of drug-resistant organisms	76	15	9	0	2.03 +/-0.9
To assess the effectiveness of patients' respiratory etiquette and hand hygiene to prevent the transmission of respiratory viruses, including influenza and coronaviruses	85	13	2	0	1.78 +/- 0.74
To develop clinical and experimental models to study cross-contamination from patient-to-patient and from the environment to patients	87	9	4	0	1.66 +/-0.8
To assess the effectiveness of hand hygiene improvement to reduce the spread of noroviruses and other viruses/microorganisms of interest	80	11	9	0	1.99 +/- 0.88

To determine the importance of hand hygiene versus environmental hygiene versus the level of patient personal hygiene on microbial transmission – the respective and combined impact	76	9	15	0	1.96 +/- 1.06
**Hand hygiene in specific settings**					
To evaluate the impact of hand hygiene compliance on mortality attributable to HAI in patients admitted to the intensive care unit	85	9	4	2	1.59 +/- 0.89
To evaluate the role of hand hygiene compliance on carriage and transmission of SARS-CoV-2 and other respiratory viruses	80	13	7	0	1.89 +/- 0.88
To evaluate the role of non-compliance with hand hygiene best practices on carriage and transmission of healthcare-associated pathogens, in the context of care of Covid-19 patients	67	28	4	0	2.1 +/- 0.83
To assess the impact of hand hygiene improvement on pathogen transmission colonization/infection in long-term care and home care	96	0	4	0	1.58 +/-0.7
To assess the impact of hand hygiene improvement on pathogen transmission/colonization/HAIs in ambulatory care	82	9	9	0	1.99 +/- 0.85
To assess the impact of hand hygiene improvement on pathogen transmission/colonization/dialysis-associated infections	87	7	6	0	1.82 +/- 0.88
To develop and test the impact of suitable strategies to achieve hand hygiene improvement and integration in the workflow of a dialysis session	82	13	4	0	1.8 +/- 0.89

To identify and assess the role of patient empowerment in achieving hand hygiene practices in hemodialysis and its impact on reducing transmission/colonization/HAI	84	11	4	0	1.8 +/- 0.86
To assess the provision of targeted education on hand hygiene for anesthesiologists on the reduction of hand contamination and infection risk during anesthesiology practices	78	13	9	0	1.93 +/- 0.99
To assess the role of anesthesiologists' hand hygiene compliance and their direct implication in infections acquired during anesthesiology practices (e.g., vascular-catheter-associated bacteremia or meningitis following spinal anesthesia)	87	9	4	0	1.73 +/- 0.86
To determine the impact of improved hand hygiene compliance (both surgical hand preparation and hand hygiene practices in the surgical ward) on surgical site infection rates	82	4	13	0	1.8 +/- 1.07
To assess the effectiveness of hand hygiene improvement strategies on preventing infection acquisition in community health care settings (such as clinics, outpatient dialysis units, etc.)	84	13	2	0	1.77 +/- 0.75
**Hand hygiene in settings with limited resources**					
To develop feasible standardized methods and indicators for HAI surveillance and hand hygiene compliance monitoring for both local evaluation and international benchmarking and establish the link with HAI reduction in low-resource settings	98	2	0	0	1.32 +/- 0.51
To identify the best methods for assessing the large-scale feasibility of hand hygiene improvement strategies in low-resource settings	91	7	2	0	1.44 +/- 0.71

To identify the best methods for assessing the cost-effectiveness of hand hygiene improvement strategies in low-resource settings	93	2	4	0	1.46 +/- 0.81
To assess the impact of implementation of hand hygiene multimodal improvement strategy on hand hygiene compliance and transmission/colonization/HAI rates in low-resource settings	89	9	2	0	1.61 +/- 0.73
To determine the impact of availability of resources needed for hand hygiene (supplies, staff for hand hygiene promotion/implementation, e.g., infection prevention and control nurses) on hand hygiene compliance and transmission/colonization/HAI rates	94	4	2	0	1.51 +/- 0.67
To assess the impact of using AHBRs on nosocomial outbreaks of scabies in low-resource settings	67	17	13	2	2.08 +/- 1.15
To explore the impact of hand hygiene compliance on infectious outcomes in settings where laboratory resources are scarce and syndromic detection of infection is used as a surrogate for laboratory-based detection	80	13	6	0	1.84 +/- 0.94
**The economic impact of improved hand hygiene**					
To assess the cost-effectiveness of a multimodal improvement strategy	91	2	7	0	1.53 +/- 0.84
To develop mathematical models to explore the cost-effectiveness of different approaches to hand hygiene improvement	91	4	4	0	1.84 +/- 0.76

To explore how financial resources dedicated to hand hygiene evolve as hand hygiene improvement strategies mature and become routine in health services	78	17	4	0	1.84 +/- 0.85

Research priorities categorized into six thematic domains: system change; training and education; evaluation and feedback; reminders and communications; institutional safety climate; and the impact of hand hygiene improvement on healthcare-associated infections and antimicrobial resistance.

Appendix C. Round 2 Delphi survey results: 2023-2030 Hand Hygiene Research Agenda, including non-consensus statements.

Safety climate

Research statement	Agree (%)	Neutral (%)	Disagree (%)	Unable to rate (%)	Mean ± SD
**Safety climate/culture change**					
The role of media (mainstream and social media) in shaping/influencing an institutional safety climate and hand hygiene improvement	75	19	6	0	2.12 +/- 0.78
**Personal accountability for hand hygiene**					
The factors that influence the development (e.g., training, mentoring, attitudes, beliefs, values) of an effective hand hygiene champion	79	15	6	0	1.75 +/- 0.98
The influence of different institutional social networks on personal accountability for hand hygiene	67	31	2	0	2.06 +/- 0.82
**Patient participation and empowerment**					
The role and impact of visitors and informal caregivers in hand hygiene improvement	71	21	8	0	2.08 +/-0.9

The perceptions of service users and patients towards an institutional safety climate and its impact on hand hygiene standards	81	10	6	2	1.99 +/- 0.84
The underlying ethical considerations in the development of a patient participation/empowerment strategy as part of an overall approach to hand hygiene improvement	61	23	12	4	2.11 +/- 1.16
**Religion and tradition**					
The influence of different religions and traditions on hand hygiene improvement strategies, including as barriers and facilitators	69	21	10	0	2.3 +/- 0.87
The relationship between religious beliefs, practices and traditions and the institutional safety climate that influences hand hygiene	62	21	15	2	2.42 +/- 0.87
The influence of religion and traditions on the acceptance and use of alcohol-based hand hygiene products	52	21	25	2	2.57 +/-1.2

Evaluation and feedback

	Agree (%)	Neutral (%)	Disagree (%)	Unable to rate (%)	Mean ± SD
**Monitoring hand hygiene performance**					
Impact on individual hand hygiene performance of his/her sense of serving as a role model for hand hygiene	75	11	11	2	2.13

					+/- 0.87
The acceptability of hand hygiene monitoring apps	70	20	7	2	2.24 +/- 0.74
**Monitoring your institution (Hand Hygiene Self-Assessment Framework [HHSAF])**					
Approaches for comparing HHSAF results from healthcare facilities in countries with different socioeconomic backgrounds and resource availability	72	18	9	0	2.16 +/- 0.85

System change

	Agree (%)	Neutral (%)	Disagree (%)	Unable to rate (%)	Mean ± SD
**General system change – enabling environment**					
To assess the role of climate change on the availability of a hand hygiene infrastructure, materials and equipment and the impact on hand hygiene compliance	48	18	27	6	2.58 +/- 1.19
To assess the role of public health emergencies of national/international concern on the availability of a hand	87	6	6	0	1.73 +/- 0.83

hygiene infrastructure, materials and equipment and the impact on hand hygiene compliance					
**Hand hygiene agents**					
To determine flash points for flammability of ABHR in relation to efficacious concentrations for hand hygiene	36	12	48	3	3.12 +/- 1.02
**Reactions to hand hygiene agents**					
To measure the role of dermal and respiratory absorption rates of alcohol during the use of ABHR and the correlation with harms (acceptable level of absorption/inhalation)	39	24	30	6	2.81 +/- 1.09
**The skin microbiome**					
To develop standardized methods for determining hand contamination from a pre-determined list of touch points in clinical settings	85	15	0	0	1.63 +/- 0.73
To develop effective (including cost-effective) methods to culture laboratory-adapted as well as field strains of gastrointestinal and respiratory viruses	69	9	18	3	2.03 +/- 1.13
To study the role of 'biotics' in supporting healthy hand microbiome flora	57	15	15	12	2.41 +/- 0.89
**Dynamics of hand transmission**					

To determine the type of hand-to-surface touch, which occurs when different potentially pathogenic organisms are present and the influence on hand hygiene opportunities	57	15	24	3	2.53 +/-1.0

Communication and reminders

	Agree (%)	Neutral (%)	Disagree (%)	Unable to rate (%)	Mean ± SD
**Hand hygiene marketing approaches**					
To study the relative contribution of each component of the marketing mix (product, price, people, place) to effective hand hygiene marketing (i.e., not solely "promotions")	58	24	13	5	2.41 +/- 0.86

HAI impact

	Agree (%)	Neutral (%)	Disagree (%)	Unable to rate (%)	Mean ± SD
**Hand hygiene in specific settings**					

To evaluate the role of non-compliance with hand hygiene best practices on the carriage and transmission of healthcare-associated pathogens in the context of care of Covid-19 patients	68	21	9	2	2.28 +/- 0.79
**Hand hygiene in settings with limited resources**					
To assess the impact of using AHBRs on nosocomial outbreaks of scabies in low-resource settings	56	30	12	2	2.21 +/- 1.04

SD, standard deviation ; HAI, healthcare-associated infections.

*Italicized statements indicate research statements that did not reach consensus following the Delphi survey rounds.

Research priorities categorized into six thematic domains: system change; training and education; evaluation and feedback; reminders and communications; institutional safety climate; and the impact of hand hygiene improvement on healthcare-associated infections and antimicrobial resistance.
